# Lipid peroxidation modifies the assembly of biological membranes “The Lipid Whisker Model”

**DOI:** 10.3389/fphys.2014.00520

**Published:** 2015-01-12

**Authors:** Ángel Catalá

**Affiliations:** Facultad de Ciencias Exactas, Instituto de Investigaciones Fisicoquímicas Teóricas y Aplicadas-Centro Científico Tecnológic La Plata-Consejo Nacional de Investigaciones Científicas y TécnicasUniversidad Nacional de La Plata, Argentina

**Keywords:** lipid peroxidation, Lipid Whisker Model, membranes, PUFAs, Fluid Mosaic Model

The aim of this opinion article is to point out the basic principles that modify the assembly of biological membranes during lipid peroxidation. With this objective in mind, I describe: the structural and functional properties of membranes, the transport and diffusion of oxygen regulated by cholesterol and fatty acids; the “Lipid Whisker Model” and finally analyzed the changes induced by lipid peroxidation in membrane structure and dynamics, both at the lipid and protein level.

Several reviews have appeared in recent years related to the kinetics and biology of lipid peroxidation products (Catala, 2010; Yin et al., 2011; Pinchuk and Lichtenberg, 2014; Vigor et al., 2014; Davies and Guo, 2014). The analysis of lipid peroxidation products has been particularly important in the advancement of research in this field because of the complexity of product mixtures. I also discuss the effect of other membrane modifications, triggered by lipid peroxidation products and reducing sugars. Contrary to what is expected by the LWM, Garrec et al. (2014) have recently investigated the validity of the “floating peroxyl radical” hypothesis by means of molecular modeling and predicted that the peroxyl radical does not “float” at the surface of the membrane.

## Introduction

When the Fluid Mosaic Model (FMM) of biological membrane structure was introduced 42 years ago, it was visualized as a basic model for cell membranes that could explain existing data on membrane proteins and lipid structures and their dynamics. According to the (FMM), a membrane was described as a biological fluid of proteins and lipids oriented in two dimensions. The basic structure of all cell and organelle membranes is the lipid bilayer. Protein molecules are distributed in different regions of the bilayer and perform diverse functions. Cell membranes are active, fluid structures, and most of their molecules are able to travel in the plane of the membrane (Singer and Nicolson, 1972). The viscosity of a lipid membrane largely depends on whether the acyl chains attached to glycerophospholipids are grouped into a rigid state or exist in a relatively disordered, fluid state. Long chain saturated fatty acids maximize Van der Waals forces, and increase the viscosity of the membrane (Chapman and Benga, 1984). Fluidity is defined as the ease of movement and represents the reciprocal of the viscosity of the membrane (Lee, 1991). The fluid properties of biological membranes are critical for various cell functions. Still slight changes in membrane fluidity may cause unusual function and pathological processes (Garcia et al., 2005). After more than 40 years, the (FMM), described by Singer and Nicolson is still relevant to recognize the structure, function, and dynamics of biological membranes (Nicolson, 2014). However, several biological processes cannot be explained on the basis of this typical phospholipid orientation and utilize other phospholipid conformations (Catala, 2012) that are described in this article.

## The structural and functional properties of membranes are determined by polyunsaturated fatty acids

The fluid properties in biological membranes are recognized principally by the presence of polyunsaturated fatty acids (PUFAs) in phospholipids molecules located in both sites of the lipid bilayer. The nature and saturation of the attached fatty acid of the phospholipids generate spectacular effects on membrane packing and fluidity (Janmey and Kinnunen, 2006). The unsaturated fatty acids give a high degree of conformational flexibility to the unsaturated hydrocarbon chains in the membranes because they occupy a small wedge-shaped space. This generally results in looser packing and a more fluid membrane. In contrast, saturated fatty acids confer rigidity that results in a less fluid or more arranged membrane. The rigidity permits saturated fatty acids to group together tightly and forms a solid at inferior temperatures. When a new double bond is open in the fatty acid produces a “kink” in the molecule. For this reason unsaturated fatty acids, such as docosahexaenoic acid (22:6 n-3), assume numerous patterns since this fatty acid can turn around in the area of C–C bonds but not in the region of the rigid C=C bonds (Feller et al., 2002). Adjustments in the lipid composition modify the fluidity of membranes. Lipid composition is modified during regulation of de novo synthesis at selected cellular places, distribution or transfer to new sites, and by specific modifying reactions. Stearyl CoA desaturase is in charge for the formation of monoethylenic fatty acids from saturated fatty acids by catalyzing the addition of a double bond into the ninth carbon of saturated C16:0 and C18:0 substrates (Enoch et al., 1976).

## The transport and diffusion of oxygen in membranes is regulated by cholesterol and fatty acids

Lipid peroxidation and formation of reactive oxygen species are important chemical reactions that use oxygen and occur in cell membranes. The diffusion of oxygen into synthetic membranes prepared with phosphatidylcholine and cholesterol have been investigated by Subczynski et al. (1991). Oxygen permeability through the membrane in all the membranes studied in this work was limited by the presence of cholesterol. Thus, they showed an increase in oxygen transport in the central part of the synthetic membranes because cholesterol decreases oxygen transport in and around the head group regions, where the main obstacles to the oxygen permeability exist. Based on this explanation, it can be assumed that non-raft areas rich in PUFA-phospholipids and vitamin E will be more available by oxygen than lipid rafts areas containing sphingolipids and cholesterol. This condition will make several micro areas more vulnerable to lipid peroxidation than others.

## The “lipid whisker model” is suitable to explain the structure of oxidized cell membranes

**Figure 1:**
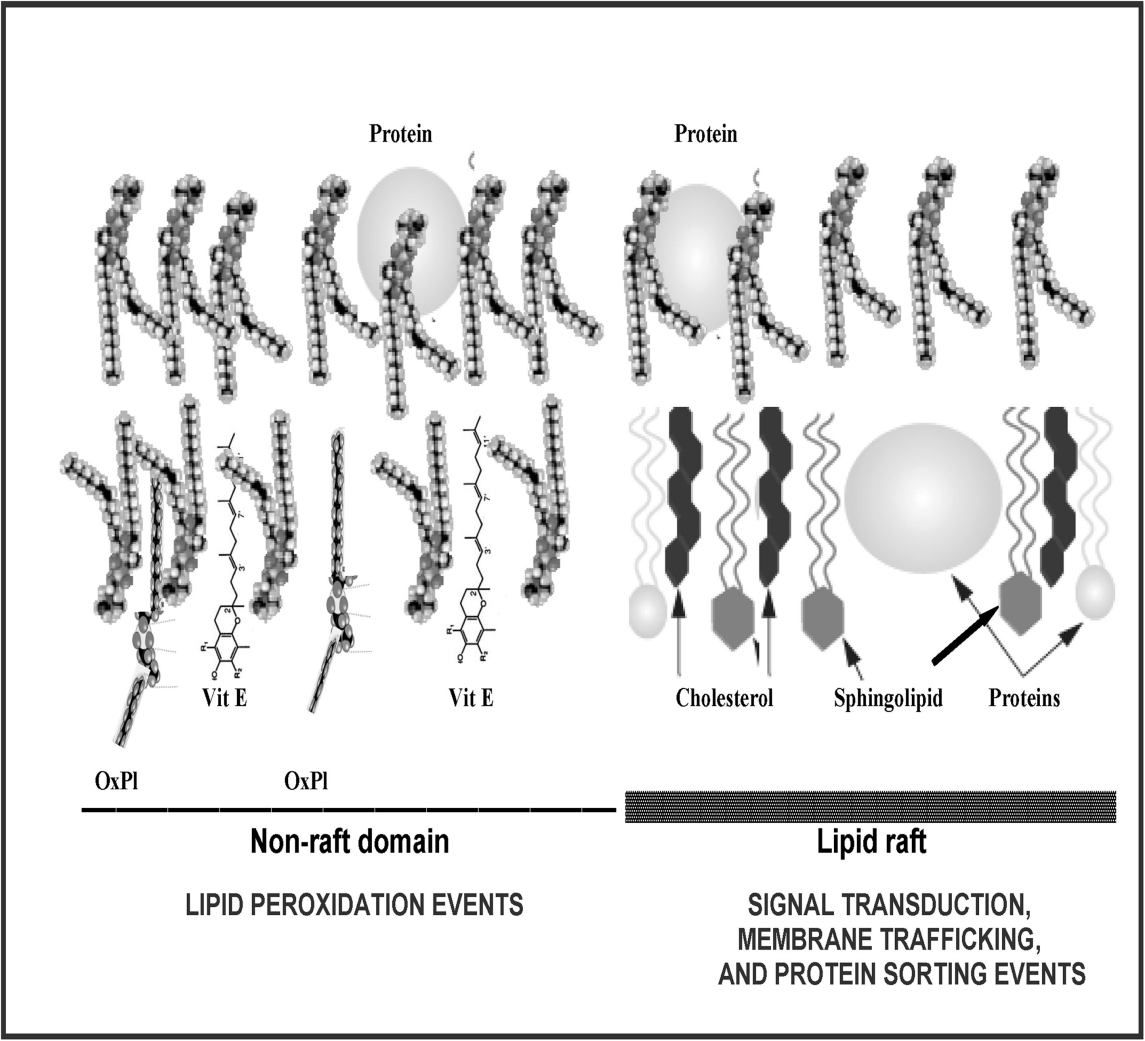
**Hypothetical model of the plasma membrane**. In terms of lipids, the heterogeneous membrane is believed to consist of a mixture of a dispersed “lipid raft” phase, enriched in cholesterol, raft-associated proteins, and saturated lipids (such as sphingolipids), and the “non-raft” matrix phase enriched with phospholipids containing PUFAs and vitamin E. Vitamin E, partition into domains that are enriched in polyunsaturated phospholipids increasing the concentration of the vitamin in the place where take place the lipid peroxidation process and oxidized phospholipids (oxPl) are formed. Reproduced from Catala (2012). Copyright © 2012 Elsevier Masson SAS. All rights reserved.

The Lipid Whisker Model (LWM) (Greenberg et al., 2008) is an extension of the Fluid Mosaic Model suggested by Singer and Nicolson (1972). Recent studies into the conformation of oxidized phospholipid (oxPL) species recognized by CD36 within model membranes (Li et al., 2007) have led to the development of the LWM. A primary attribute of the (FMM) is that amphipathic phospholipids are oriented in an organization lamellar mesophase with hydrophobic fatty acyl chains embedded within the interior of the membrane and the hydrophilic polar groups facing the aqueous environment. This lipid organization accepts fast lateral circulation of lipids and membrane proteins similarly within the planar membrane surface. It also causes the water-resistant character of cell membranes to hydrophilic molecules. But, recent information proposes that in peroxidized cell membranes, numerous of the oxPL classes adopt a particular conformation. Lipid peroxidation is achieved by addition of numerous polar molecules on fatty acid chains (Catala, 2009). Consequently, when cell membranes undergo oxidation, if not adapted by the action of phospholipases, they may “produce whiskers” including a variety of oxidized sn-2 fatty acids of diverse structures. In the (LWM), the assembly of many oxPL within cell membranes is different compared with one observed in non-oxPL described in the (FMM). Biophysical studies have shown that the addition of an oxygen atom to the acyl chain produces a significant change that prevent its immersion in the interior of the membrane, however, the modified acyl chain is projected on the aqueous medium (Figure 1) (Subczynski et al., 1991; Greenberg et al., 2008).

As cell membranes are peroxidized “grow whiskers” because phospholipids are peroxidized, and several of its oxidized fatty acids are projected onto the surface (Figure 1). This change produced by the oxidation of a fatty acid chain in the membrane may be a trigger event to numerous biological activities.

## The effect of other membrane modifications, triggered by lipid peroxidation products and reducing sugars introduces changes in cell membrane physico-chemical and biological properties

During lipid peroxidation, biomolecules such as proteins or amino lipids can be covalently modified by lipid decomposition products (Catala, 2009). For the case of aliphatic aldehydes (alkanals) such as 1-hexanal or 1-nonanal, the Nε-amino groups of the lysine residues in proteins can be modified through the formation of a Schiff base. α,β-Unsaturated aldehydes (alkenals) such as acrolein or 4-hydroxy-2-nonenal react with lysine, cysteine, and histidine through a Michael-type addition. Conversely, lipid hydroperoxide might covalently react with protein without serious decomposition of its structure. However, the mechanism of lipid hydroperoxide-derived protein modification is not so clear. Evidence for in situ ethanolamine phospholipid adducts with hydroxy-alkenals has been recently described.

Non-enzymatic modification of aminophospholipids by lipid peroxidation-derived aldehydes and reducing sugars through carbonyl-amine reactions are thought to contribute to the age-related deterioration of cellular membranes (Naudí et al., 2013). Much evidence demonstrates the modification of aminophospholipids by glycation, glycoxidation and lipoxidation reactions.

## Lipid peroxidation in membranes: the peroxyl radical does not “float”

Some key microscopic aspects of the lipid peroxidation reaction in cell membranes are still poorly studied. In particular, it is usually accepted that the propagation of the radical reaction in lipid bilayers is hampered by the rapid diffusion of peroxyl intermediates toward the water interface, that is, out of the reaction region. Contrary to what is expected by the LWM, Garrec et al. (2014) have recently investigated the validity of this “floating peroxyl radical” hypothesis by means of molecular modeling. Combining quantum calculations of model systems and atomistic simulations of lipid bilayers containing lipid oxidation products, these authors predict that the peroxyl radical does not “float” at the surface of the membrane. Instead, it remains located quite deep inside the bilayer.

## The changes induced by lipid peroxidation in membrane structure and dynamics, both at the lipid and protein level

The mechanisms and principles establishing the thousands of proteins and lipids that make up membrane bilayers in cells are still unclear. Mueller et al. reviewed the basic properties of biological membranes and the most common theories for lateral segregation of membrane components before discussing an emerging model of a self-organized, multi-domain membrane or “patchwork membrane” (Mueller et al., 2012).

The oxidation of phospholipids has become a recent topic of interest within the field of membrane biophysics. Still, the exact mechanism of membrane injury by oxidized lipids is uncertain (Wong-Ekkabut et al., 2007).

Membrane rafts remain one of the most controversial issues in biophysics, because the methods for their detection are still far from perfect. Optical techniques rely heavily on fluorescent markers. In biological membranes, these probes are connected with lipids and thus define only the lipid part of the membrane. Therefore, they are “blind” to the presence of membrane proteins, unless FRET techniques between proteins and membrane probes are used. Therefore, the imperative future challenge for the membrane probes will be to design molecules capable of monitoring lipid surrounding specifically around a given protein of interest (Klymchenko and Kreder, 2014).

Proteins and lipids in membrane processes are reciprocally dependent on each other. While the lipid domains as organizers of proteins has attracted wide-spread consideration, the question whether proteins control lipids or lipids control proteins in cell membranes is not a simple problem to solve. The fact that proteins exploit the biophysical properties of lipids including lipid charge and phase separation for membrane function is one of the reasons why membrane biology is such an attractive area of research (Rossy et al., 2014).

To guarantee coordination of cellular activities, cells use membrane contact sites (MCSs) between the membranes of diverse organelles. MCSs are domains where two membranes come to close proximity, typically less than 30 nm, and create microdomains that favor exchange between two organelles. Since the endoplasmic reticulum (ER) is the most widespread cellular membrane network, it is thus not surprising to find the ER involved in most MCSs within the cell. The ER contacts diverse compartments such as mitochondria, lysosomes, lipid droplets, the Golgi apparatus, endosomes, and the plasma membrane. Taken into account these observations, several levels of complexity have to be considered when analyzing the changes induced by lipid peroxidation in membrane structure and dynamics, both at the lipid and protein level.

In my opinion and in the light of new investigations, several critical aspects of lipid peroxidation in biological membranes, such as their assembly and structural organization need to be revisited.

### Author note

The author is a member of Carrera del Investigador Científico, Consejo Nacional de Investigaciones Científicas y Técnicas (CONICET), Argentina.

### Conflict of interest statement

The author declares that the research was conducted in the absence of any commercial or financial relationships that could be construed as a potential conflict of interest.

## Abbreviations

22:6 n-3: docosahexaenoic acid
ER: endoplasmic reticulum
CD36: fatty acid translocase
FMM: Fluid Mosaic Model
FRET: Förster Resonance Energy Transfer
LWM: Lipid Whisker Model
oxPL: oxidized phospholipid
16:0: palmitic acid
PC: phosphatidylcholine
PUFAs: polyunsaturated fatty acids
18:0: stearic acid.
